# Antagonistic Activity of Anandamide on Tat-treated Human Astrocytes Identifies Inflammaging Pathways: Anandamide Affects Aging Pathways

**DOI:** 10.18502/jovr.v21.17666

**Published:** 2026-07-14

**Authors:** Durairaj Duraikannu, Kamini Khatak, Hemavathy Nagarajan, Sridharan Sudharshan, Nivedita Chatterjee

**Affiliations:** ^1^L&T Department of Ocular Pathology, Vision Research Foundation, Chennai, Tamil Nadu, India; ^2^Centre for Biotechnology, Anna University, Chennai, Tamil Nadu, India; ^3^Ion Channel Biology Laboratory, AU-KBC Research Centre, Madras Institute of Technology, Anna University, Chennai, Tamil Nadu, India; ^4^Department of Bioinformatics, Vision Research Foundation, Chennai, Tamil Nadu, India; ^5^Department of Uveitis, Medical Research Foundation, Chennai, Tamil Nadu, India; ^6^The manuscript was archived and posted on bioRxiv: https://biorxiv.org/cgi/content/short/2024.10.21.619556v1

**Keywords:** Anandamide, Endocannabinoid, Human Immunodeficiency Virus, Inflammaging, Senescence

## Abstract

**Purpose:**

The endocannabinoid system can suppress inflammatory environment by regulating inflammatory mechanisms in immune and glial cells. Astrocytes secrete soluble inflammatory mediators. Prolonged activation of astrocytes is associated with accelerated aging in the central nervous system. MicroRNAs are increasingly shown to be critical gene regulators during inflammation and gliosis. In this study, we investigated the microRNA changes affected by anandamide (AEA), a dominant endocannabinoid in normal human astrocytes, following exposure to the HIV-1 Tat (Trans-activator of transcription) protein.

**Methods:**

We performed global human microRNA profiling in Tat-activated astrocytes on exposure to AEA. To delineate the mechanism of action, we utilized the bioinformatic tools miRWalk, KEGG, and Cytoscape to assess the global microarray data for significantly impacted miRNAs and their gene targets at the mRNA level.

**Results:**

Tat-induced activation significantly upregulated 122 miRNAs (*P* < 0.05) in astrocytes. Conversely, the addition of AEA in activated astrocytes significantly downregulated the expression of 57 miRNAs. Out of 122 miRNAs upregulated by Tat treatment, 37 miRNAs that were common to Tat and Tat+AEA cells showed reversed expression, suggesting these might be the critical miRNAs with a key role in the AEA-induced mitigation of neuroinflammation.

**Conclusion:**

Reversed expression of a selected group of miRNAs identifies antagonistic pathways that promote an anti-inflammatory environment. Pathway analysis of these 37 key miRNAs showed gene targets that regulate inflammation and senescence.

##  INTRODUCTION

The endogenous cannabinoid system modulates immune phenomena associated with infection or inflammation. Due to their ability to suppress lymphocyte proliferation and inflammatory cytokine production, they are regarded as promising therapeutic targets for diseases with an immune phenotype.^[1]^ Because all immune cells examined so far express cannabinoid receptors regardless of their cell lineage,^[2]^ all types of immunity are sensitive to cannabinoid modulation. Endocannabinoid (EC) modulation of immune reactions has been studied extensively in microglia,^[3]^, astrocytes,^[4]^ and oligodendrocytes.^[5]^ At the retina, which is an extension of the central nervous system (CNS), Müller glia comprise the largest glial class followed by astrocytes. Two ECs, anandamide (AEA), and 2-arachidonoylglycerol (2-AG) modulate the innate immune response of the retinal Müller glial cells.^[6,7]^ We have previously shown that AEA and 2-AG reduce inflammation by targeting key inflammatory machinery at multiple signaling levels in retinal Müller glia.

Studies on macaques infected by Simian immunodeficiency virus (SIV) upon Δ THC administration and investigations on people with human immunodeficiency virus (HIV) using cannabis have also shown decreased levels of T-cell activation, inflammatory monocytes, and pro-inflammatory cytokine secretion.^[8]^ We aimed to investigate the effects of endogenous cannabinoids on HIV-1 activation in astrocytes. Despite viral suppression by anti-retroviral therapy (ART), the symptoms of HIV-associated neurocognitive disorder (HAND) persist in the CNS. In the brain, instead of residing in T-lymphocytes, the virus persists in astrocytes and microglia, establishing a reservoir of infection. Some HIV proteins, including secreted ones such as the HIV-1 trans-activator of transcription (Tat), can induce inflammation in cells and, thereby, cause neurodegeneration. Persistent chronic inflammation and its underlying molecular mechanisms can lead to many changes, including premature aging.

MicroRNAs (miRNAs) are small noncoding RNAs (21-25 nucleotides long) that regulate gene expression by degrading or silencing target mRNA molecules via complementary seed sequence binding. miRNAs bind to the 3' untranslated region (3'UTR) or 5'UTR of their target mRNAs. The complexity of these interactions increases with the presence of numerous binding sites per miRNA and the potential of each mRNA to be targeted by multiple miRNAs.^[9,10]^

In this study, we first established an activated immune state with HIV-1 Tat in cultured human astrocytes and investigated the effects of AEA. We looked at the global changes in miRNAs upon AEA treatment, which is involved in the regulation of inflammation, particularly in the context of inflammaging. The EC AEA has been shown to downregulate key miRNAs that attenuate inflammation in a *Staphylococcus* *enterotoxin* B model of acute respiratory inflammation by suppressing immunosuppressive T regulatory cells.^[11]^

In a global miRNA microarray analysis, the addition of Tat to astrocytes significantly increased the expression of 122 miRNAs, many of which are related to inflammation. We analyzed the anti-inflammatory role of AEA in the Tat-stimulated astrocytes. AEA addition, along with Tat, reversed the expression of 37 miRNAs (out of 122 miRNAs in Tat-treated cells), suggesting their involvement in systematic suppression and their potential as therapeutic targets for pharmacological intervention.

##  METHODS

### Primary Cell Culture and Treatment

The Ethics Committee at the Vision Research Foundation (Ethics 358-2013-P), Chennai, India, approved this study. Normal human astrocytes (Cat# CC-2565, Lonza) were cultured with AGM Astrocyte Growth Medium Bullet Kit (Basal Medium and SingleQuots Kit; Cat# CC-3186) for cell-specific growth. Cells were seeded into a T75 culture flask and allowed to grow in a CO ${}_{2}$ incubator for a week to attain 80% confluency [Supplementary Figure 1]. The cells were plated into 6-well plates for treatment. Once the cells reached 80% confluency, they were treated with HIV-1 Tat recombinant protein, AEA, or Tat+AEA for 24 hours, and then collected for microarray. No morphological changes were observed in the cells within 24 hours after treatment. HIV-1 Tat recombinant protein (100 ng/mL) was purchased from NIH AIDS Reagent Program (ARP 2222). Activation of treated cells was tested by measuring inflammatory cytokines [Supplementary Figure 1]. The lyophilized powder was reconstituted with sterile PBS reagent, and AEA (Sigma-Aldrich) was reconstituted in DMSO to attain a working concentration of 10 μ M.

### RNA Isolation 

Treated cells were harvested with TRIzol Reagent (Cat. No: 15596018, Invitrogen). Phase separation was induced by the addition of chloroform. The clear upper aqueous phase was collected, and the RNA was precipitated using isopropanol. Next, isopropanol was removed using 70% ethanol. The precipitate was air-dried and dissolved in RNase-free water.

### Global Microarray Profiling

Global human miRNA microarray profiling was performed by using Agilent's Complete miRNA Labeling and Hybridization Kit on Human miRNA Microarray slides (8X60k AMADID: 70156; Cat # 5190-0456). Expression of miRNAs is presented as log-2fold change. The cut-off value was 0.6. Additionally, the miRNA expression levels of Tat- and AEA-treated samples were compared with and normalized to those in untreated astrocytes (control). The miRNA expression levels of Tat+AEA-treated samples were normalized to Tat-treated astrocytes using GeneSpring GX software (Agilent Technologies, 2016).

Normalization to specific samples: Treated vs Control. Percentile shift normalization is a global normalization where the intensities of all spots on an array are adjusted. This normalization analyzes each column in an experiment independently and computes the percentile of expression values across all spots in the array, where n ranges from 0 to 100, and n = 50 is the median. This value is subtracted from the expression value of each entity.

### miRNA–mRNA Network

In our study, the addition of Tat increased the expression of 122 miRNAs. When AEA and Tat are added together, the expression of 37 miRNAs (out of 122 miRNAs) is reversed. These differentially expressed miRNAs were further analyzed using the miRWalk database (http://mirwalk.umm.uni-heidelberg.de/) to predict their target messenger RNAs. Functional mRNAs with high binding energy (cut-off set as 1) were shortlisted. Cytoscape software (https://cytoscape.org) was then used to understand interactions between the shortlisted miRNAs and their predicted mRNAs.

### Pathway Enrichment Analysis

Shortlisted mRNAs were analyzed in KOBAS (KEGG Orthology-based Annotation System; https://bio.tools/kobas) to predict pathways regulated by the miRNAs. By keeping the adjusted P-value < 0.05, significant pathways that are related to inflammation and aging were identified.

### Protein–Protein Interaction (PPI) Network Analysis

The Search Tool for the Retrieval of Interacting Genes (STRING; http://string-db.org/) was adopted to demonstrate the interactions between proteins that are predicted as the targets of the shortlisted miRNAs.

### Comparative Analysis of the Target miRNAs 

A comparative analysis was carried out between the resultant miRNA–mRNA interactions and the known mRNAs of genes that are identified in physiological processes such as the inflammasome, senescence, and the EC system. These were compared with the miRNAs experimentally observed in our study. The resultant mRNAs were analyzed using the MicroRNA Target Prediction Database (miRDB; http://mirdb.org) to predict their regulatory miRNAs. Subsequently, the miRNAs common to both miRDB predictions and our microarray profile were reported.

**Figure 1:**
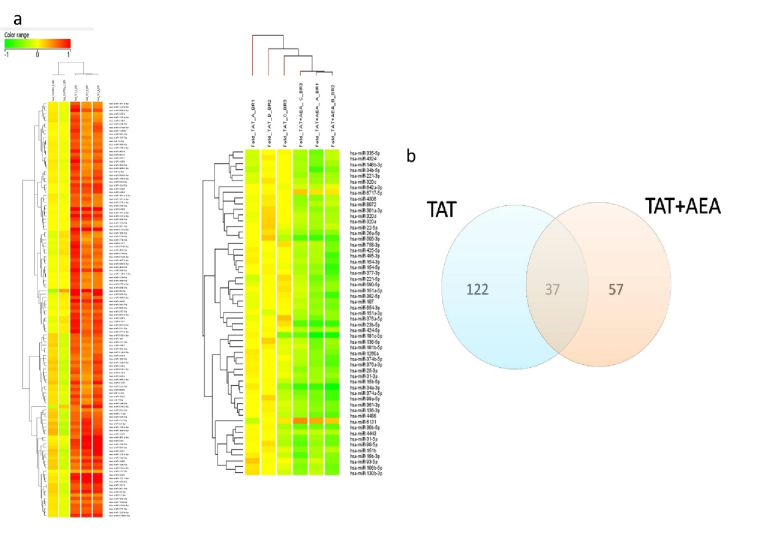
(a) AEA alters miRNA expression in human astrocytes treated with HIV-1 Tat coat protein. Primary human astrocytes were screened for microRNA expression as described in Methods. The heat map shows miRNAs altered following Tat and Tat+AEA treatment. Red denotes upregulation and green denotes downregulation. Fold change range is taken between –1 and 1. (b) Tat treatment in astrocytes significantly increases the expression of 122 miRNAs. Tat+AEA decreases the expression of 57 miRNAs. Out of 122 upregulated miRNAs, 37 miRNAs are downregulated by AEA. Normalized to specific samples: Treated vs Control Percentile Shift Normalization is a global normalization, where the locations of all the spot intensities in an array are adjusted. This normalization takes each column in an experiment independently and computes the percentile of the expression values for this array across all spots (where *n* has a range from 0 to 100 and *n* = 50 is the median). It subtracts this value from the expression value of each entity.

##  RESULTS 

### miRNA Profiling

**Figure 2:**
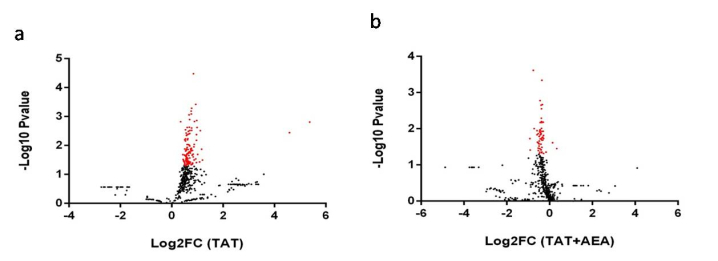
(a) Volcano plot representing the expression of miRNAs in Tat-treated astrocytes. The majority of miRNAs are upregulated with Tat. miRNAs that are significantly upregulated are represented in red. (b) Volcano plot representing the expression of miRNAs in Tat+AEA-treated astrocytes. The majority of miRNAs are downregulated with Tat+AEA. miRNAs that are significantly downregulated appear in red.

The miRNA expression is mainly regulated at the level of transcription. HIV-1 Tat regulates the transcription of host genes and miRNAs through several transcription factors, including CREB.^[12]^ The role of miRNAs in regulating the aging and inflammatory processes has been studied before.^[13]^ Figure 1(a) presents miRNA expression upon Tat and Tat+AEA treatment as a heat map generated using GeneSpring GX software. Treating astrocytes with Tat protein upregulated 470 miRNAs, of which 122 miRNAs showed significant values (*P* < 0.05) [Figures 1b–c & 2a]. The fold change values of upregulated miRNAs ranged between 0.5 and 5.37. Specifically, hsa-miR-8485, hsa-miR-513a-5p, hsa-miR-96-5p, hsa-miR-543, and hsa-miR-1271-5p were highly upregulated. Significant upregulation of 122 miRNAs confirms the regulation of many mRNAs controlled by these miRNAs. In our data, Tat downregulated two miRNAs: hsa-miR-4701-5p and hsa-miR-6763-3p.

Exposure to AEA increased the expression of 266 miRNAs compared to untreated controls. Out of 266 miRNAs, 52 miRNAs were significantly upregulated. This was statistically significant in regard to hsa-miR-4788, hsa-miR-8485, hsa-miR-4698, hsa-miR-6500-5p, and hsa-miR-6777-3p. AEA treatment led to only one downregulated miRNA; hsa-miR-145-3p.

Out of 386 miRNAs modified upon Tat+AEA treatment, 57 miRNAs were significantly downregulated compared to cells treated with Tat alone [Figure 2b]. Downregulation of these 57 miRNAs indicates the role of AEA in reversing the expression of multiple regulatory elements. It should be noted that the miRNAs, including hsa-miR-8485, hsa-miR-513a-5p, hsa-miR-96-5p, hsa-miR-543, and hsa-miR-1271-5p, which were upregulated by Tat, were not downregulated by Tat+AEA. Additionally, Tat+AEA-treated astrocytes showed upregulation in seven miRNAs (hsa-miR-6768-5p, hsa-miR-6780a-5p, hsa-miR-6880-3p, hsa-miR-2116-3p, hsa-miR-6512-5p, hsa-miR-3125, and hsa-miR-6777-3p), none statistically significant. Strikingly, AEA has an antagonistic effect in Tat-activated cells, but not in resting cells.

We further investigated the statistically significant miRNAs. Since the purpose was to examine the effect of AEA in the presence of Tat, we focused mainly on miRNA expression in Tat+AEA-treated cells. Out of 57 miRNAs downregulated by Tat+AEA treatment, 37 miRNAs were common between Tat (122 upregulated miRNAs) and Tat+AEA treatment. Therefore, AEA reversed the expression of a large number of miRNAs that were upregulated by Tat upon immune activation. KEGG enrichment analysis was carried out for the target genes of these 57 miRNAs. These miRNAs are associated with the PI3-Akt signaling pathway, the Ras signaling pathway, the MAPK signaling pathway, the longevity regulating pathway, the TNF signaling pathway, and pathways related to cancer.

### Target Prediction

We performed bioinformatic analysis on 37 miRNAs that were upregulated by Tat treatment and downregulated by AEA in Tat-activated cells. Based on the results, these miRNAs play a significant role in the antagonistic effects of AEA on Tat. The total number of target mRNAs predicted for each miRNA is given in Supplementary Table 1 (high binding energy cut-off set as 1). Only mRNAs that are regulated by more than one miRNA [Tables 1 & 2] were considered for generating the miRNA-mRNA networks.

**Figure 3:**
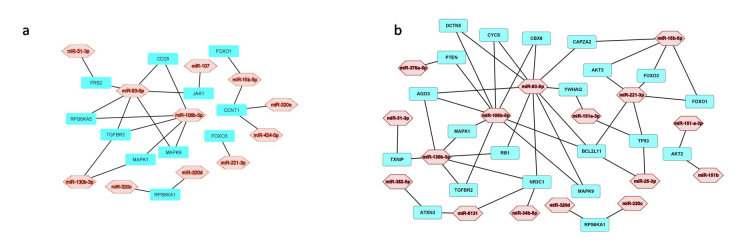
The network image shows the relationship between miRNAs and target mRNAs that were downregulated on Tat+AEA treatment. The network image of (a) inflammasome-related miRNA–mRNA interactions and (b) senescence-related miRNA–mRNA interactions. Functional mRNAs with high binding energy (cut-off set as 1) were shortlisted.

We also found that miR-15b-5p, miR-93-5p, and miR-106b-5p were predicted to regulate many genes that are associated with inflammation and senescence. miR-15b-5p, miR-93-5p, and miR-106b-5p were upregulated with fold changes of 0.63, 0.76, and 0.79, respectively, following Tat treatment. On Tat+AEA treatment, these were downregulated with fold changes of –0.44, –0.36, and –0.45, respectively. Significant predicted mRNAs associated with the inflammatory machinery included FOXO1, FOXO3, MAPK1, MAPK9, JAK1, FRS2, RPS6KA5, and RPS6KA1. The corresponding miRNA–mRNA regulatory network is illustrated in Figure 3a. Significant predicted mRNAs related to senescence pathways were FOXO1, FOXO3, NR3C1, ATXN3, CAPZA2, AKT3, BCL2L11, TP53, TXNIP, CYCS, MAPK9, AGO3, DCTN5, RB1, CBX8, TGFBR2, MAPK1, PTEN, YWHAQ, AKT2, and RPS6KA1, and their miRNA–mRNA network is shown in Figure 3b.

### Pathway Enrichment Analysis

**Figure 4:**
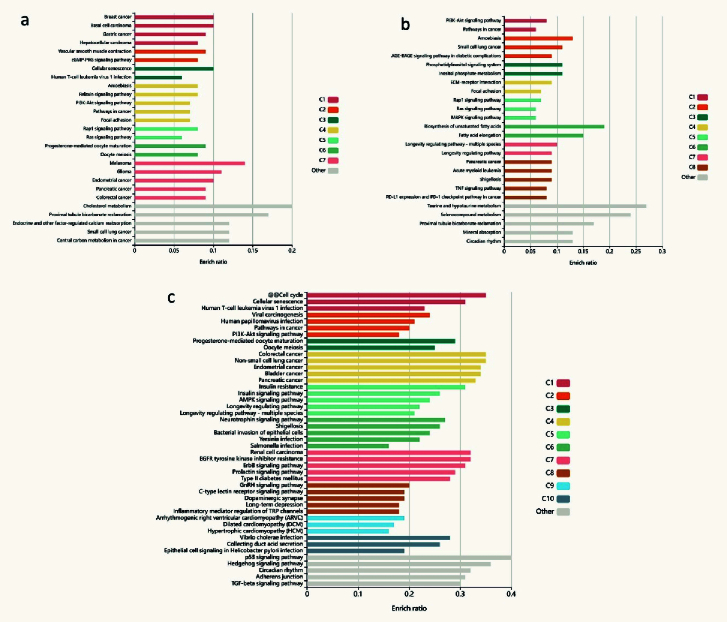
(a) KEGG pathway enrichment analysis was performed for mRNAs predicted as targets for 122 miRNAs upregulated following Tat treatment. (b) KEGG pathway enrichment analysis was performed for mRNAs predicted as targets for 57 miRNAs downregulated following Tat+AEA treatment. (c) KEGG pathway enrichment analysis was performed for mRNAs predicted as targets for 37 downregulated miRNAs common to TAT and TAT+AEA treatments. In the enrichment graph, each row represents enriched function, and the length of the bar represents the enriched ratio. Colored bars represent different clusters. Considering an adjusted *P*-value of < 0.05, significant pathways related to inflammation and aging were identified.

Genes predicted by miRWalk were used for KEGG pathway enrichment analysis [Figure 4a–c]. Significant pathways predicted by KEGG were related to the cell cycle, various cancer pathways, TNF pathway, cellular senescence, viral infection, PI3K–Akt pathway, HIV-1 infection, and the TGF beta pathway.

### Protein–Protein Interaction (PPI) Network Analysis

**Figure 5:**
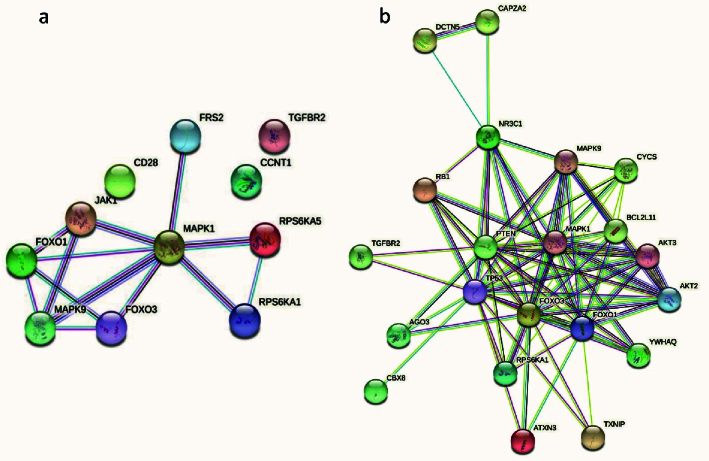
The STRING network shows protein–protein interactions among target genes regulated by more than one shortlisted miRNA. STRING network images illustrating (a) inflammasome-related genes and (b) senescence-related genes.

We constructed a PPI network using the STRING database (https://www.string-db.org/) to identify interacting genes/proteins. Using the PPI network, we identified interactions between inflammatory proteins that were translated from mRNAs within the miRNA network. FOXO1, FOXO3, MAPK1, MAPK9, JAK1 FRS2, RPS6KA5, and RPS6KA1 showed significant interconnections [Figure 5a]. The STRING network showed that the MAPK1 enzyme acts as a junction protein connecting many of the other shortlisted proteins.

Analysis of interactions between senescence-related mRNAs identified FOXO1, FOXO3, NR3C1, ATXN3, CAPZA2, AKT3, BCL2L11, TP53, TXNIP, CYCS, MAPK9, AGO3, DCTN5, RB1, CBX8, TGFBR2, MAPK1, PTEN, YWHAQ, AKT2, and RPS6KA1 [Figure 5b].

**Table 1:** KEGG pathway enrichment analysis of mRNAs regulated by more than one shortlisted miRNA for inflammasome-related genes

**Sr. No.**	**miRNAs**	**Predicted targets (mRNAs)**
1	miR-15b-5p, miR-320e, miR-424-5p	CCNT1
2	miR-93-5p, miR-106b-5p	CD28
3	miR-15b-5p, miR-221-3p	FOXO1, FOXO3
4	miR-31-3p, miR-93-5p	FRS2
5	miR-93-5p, miR-107	JAK1
6	miR-106b-5p, miR-130b-3p, miR-93-5p	MAPK1, MAPK9
7	miR-320c, miR-320d	RPS6KA1
8	miR-93-5p, miR-106b-5p	RPS6KA5
9	miR-93-5p, miR-106b-5p, miR-130b-3p	TGFBR2

**Table 2:** KEGG pathway enrichment analysis of mRNAs regulated by more than one shortlisted miRNA for senescence-related genes

**Sr. No.**	**miRNAs**	**Predicted targets (mRNAs)**
1	miR-15b-5p, miR-221-3p	FOXO1, FOXO3
2	miR-6131, miR-34b-5p, miR-93b-5p, miR-130b-3p	NRC1
3	miR-6131, miR-382-5p	ATXN3
4	miR-15b-3p, miR-93-5p	CAPZA2
5	miR-15b-3p, miR-221-3p	AKT3
6	miR-221-3p, miR-25-3p, miR-93-5p, miR-106b-5p	BCL2L11
7	miR-151a-3p, miR-25-3p, miR-221-3p	TP53
8	miR-93-5p, miR-130b-5p	TXNIP
9	miR-93-5p, miR-106b-5p	CYCS
10	miR-93-5p, miR-106b-5p	MAPK9
11	miR-93-5p, miR-106b-5p, miR-130b-3p	AGO3
12	miR-93-5p, miR-106b-5p	DCTN5
13	miR-93-5p, miR-130b-5p	RB1
14	miR-93-5p, miR-106b-5p	CBX8
15	miR-106b-5p, miR-130b-5p	TGFBR2
16	miR-106b-5p, miR-130b-5p	MAPK1
17	miR-106b-5p, miR-376a-5p	PTEN
18	miR-93-5p, miR-151a-3p	YWHAQ
19	miR-151a-3p, miR-151b	AKT2
20	miR-320c, miR-320d	RPS6KA1

### Comparative Analysis of the Target miRNAs 

Our study was designed to examine miRNAs and their target genes on treatment with EC AEA in Tat-stimulated human astrocytes. As part of a comparative analysis of our microarray data, we predicted miRNAs targeting mRNAs that were involved in inflammasome (GSDMD, NLRP1, CARD8, IL-1 α , IL1- β , IL-18, AIM2, and NLRX1), senescence (SIRT1, FOXO3, FOXO4, and CDKN2A), and EC system genes (FAAH, NAPEPLD, MGLL, and CNR1). *In silico* prediction of miRNAs by miRDB for these signaling pathway components showed commonalities in miRNAs that were identified in our experimental astrocyte data.

Supplementary Table 2 lists Tat- and Tat+AEA-regulated miRNAs that are predicted by miRDB for target gene prediction and functional annotations. miRNAs that appeared in both our microarray and miRDB predictions for inflammasome-related genes include hsa-miR-106b-5p, hsa-miR-93-5p, hsa-miR-495-3p, hsa-miR-4306, hsa-miR-15b-5p, and hsa-miR-424-5p. Common miRNAs of the EC system were hsa-miR-106b-5p, hsa-miR-151a-3p, hsa-miR-15b-5p, hsa-miR-34b-5p, hsa-miR-424-5p, and hsa-miR-93-5p. Furthermore, common miRNAs implicated in senescence were hsa-miR-154-3p, hsa-miR-6131, hsa-miR-361-3p, hsa-miR-4306, hsa-miR-495-3p, and hsa-miR-590-5p. This comparative analysis confirms that the miRNAs differentially regulated upon Tat and Tat+AEA treatment in our microarray study are associated with the inflammasome, senescence, and EC system.

##  DISCUSSION

Dysregulated miRNAs contribute to chronic inflammation in the brain, thereby leading to the progression of many neurological diseases. Reactive glial populations are implicated in this amplified and prolonged neuroinflammatory response.^[15,16]^ The current study was designed to investigate miRNAs involved in inflammation that are associated with the senescence phenotype and the immunomodulatory potential of the EC AEA.

Based on our findings through bioinformatic analysis, the addition of endogenous cannabinoids in Tat-treated astrocytes led to changes in multiple miRNAs, which target anti-inflammatory factors. Numerous miRNAs have been shown to be significantly up- or downregulated during aging. Many of these miRNAs, such as the miR-71 and miRNA lin-4, were first identified in *C. elegans *as critical regulators of lifespan.^[17]^ Similarly, the miR-17-92 cluster in mammals has been shown to be a regulator of aging and cellular senescence in various co-morbidities.^[18,19]^ Several studies have confirmed that endogenous cannabinoids can reduce inflammation in glial cells by controlling the signaling machinery at multiple levels. In addition, the anti-inflammatory action of AEA and agonists such as WIN-55212-2 can suppress proinflammatory factors like Interleukin 1 Beta and IL-6.^[20,21]^

Earlier work from our group identified the seminal role of the MAPK-NFk-B axis in the anti-inflammatory effects of AEA and 2-AG within Müller glia.^[5]^ AEA also restores barrier properties of Müller cells by controlling nitric oxide production.^[6]^ The neuroprotective mechanism involved suppressing the production of proinflammatory cytokines and increasing anti-inflammatory cytokines, mainly through the MAPK and PI3K–Akt pathways.^[7]^ Among the downstream mediators of the PI3K–Akt pathway, Forkhead box O (FOXO) is negatively regulated by Akt signaling.^[22]^

Taken together with our previous findings in retinal glia, this observation suggests that AEA suppresses inflammation by modulating several miRNAs that target proinflammatory cytokine pathways. Our data confirms the dynamic regulatory role of the identified miRNAs. They are likely to serve as potential mechanistic links between immunity as a central node and its causal involvement in aging.

In summary, we studied the exacerbation of functional pathways in astrocytes induced by HIV-1 Tat. To the best of our knowledge, this is the first report of AEA-mediated alterations in miRNA expression in activated primary human astroglia. The role of miRNAs and their targetome in astrocyte activation and AEA-induced changes is just beginning to be understood. Given that HIV-associated neurodegeneration is not limited to neurons, the action of endogenous cannabinoids on astrocytes—showing the regulatory role of miRNAs on phenotypes associated with cellular senescence—is indicative of alterations in cellular metabolism, secreted cytokines, epigenetic regulation, and protein expression, thereby providing important insights into modulating immune responses in the CNS.

### Supplementary Information

The microarray data discussed in this manuscript have been deposited in the NCBI Gene Expression Omnibus (GEO) under the GEO series accession number GSE210095.

### Financial Support and Sponsorship

This study was supported by the Department of Biotechnology, Government of India, BT/PR8585/AGR/36/780/2013. DD and KK are supported by the Council of Scientific and Industrial Research.

### Conflicts of Interest

None.
